# Normal vaginal delivery at term after expectant management of heterotopic caesarean scar pregnancy: a case report

**DOI:** 10.1186/s13256-018-1713-0

**Published:** 2018-06-21

**Authors:** Olga Vikhareva, Ekaterina Nedopekina, Andreas Herbst

**Affiliations:** 1 Department of Obstetrics and Gynaecology, Skåne University Hospital Malmö, Lund University, Box 117, 221 00 Lund, Sweden; 2 Department of Obstetrics and Gynaecology, Skåne University Hospital, Lund University, Jan Waldenströms gata 47, SE-20502 Malmö, Sweden

**Keywords:** Heterotopic caesarean scar pregnancy, Expectant management, Vaginal delivery

## Abstract

**Background:**

Heterotopic pregnancy with a combination of a caesarean scar pregnancy and an intrauterine pregnancy is rare and has potentially life-threatening complications.

**Case presentation:**

We describe the case of a 27-year-old white woman who had experienced an emergency caesarean delivery at 39 weeks for fetal distress with no postpartum complications. This is a report of the successful expectant management of a heterotopic scar pregnancy. The gestational sac implanted into the scar area was non-viable. The woman was treated expectantly and had a normal vaginal delivery at 37 weeks of gestation.

**Conclusion:**

Expectant management under close monitoring can be appropriate in small non-viable heterotopic caesarean scar pregnancies.

## Background

Heterotopic caesarean scar pregnancy (CSP), in which one gestational sac is located in the caesarean scar area and the other one is a normal intrauterine pregnancy, is rare and may have potentially life-threatening complications. The correct management of this condition is unclear. It is a challenge to manage a heterotopic CSP with preservation of the intrauterine pregnancy minimizing the risks for mother and child.

Transvaginal sonography is a valuable diagnostic tool in the management of such pregnancies [1]. Currently, we offer women with one previous caesarean section participation in an ongoing study of transvaginal ultrasound examinations in each trimester. The study provides support to identify patients with high risk of uterine rupture/potential placental complications to make an individual plan for pregnancy surveillance and delivery.

We have not found previous reports on successful expectant management of spontaneous heterotopic CSP with the preservation of intrauterine pregnancy resulting in a normal vaginal delivery.

## Case presentation

**Fig. 1:**
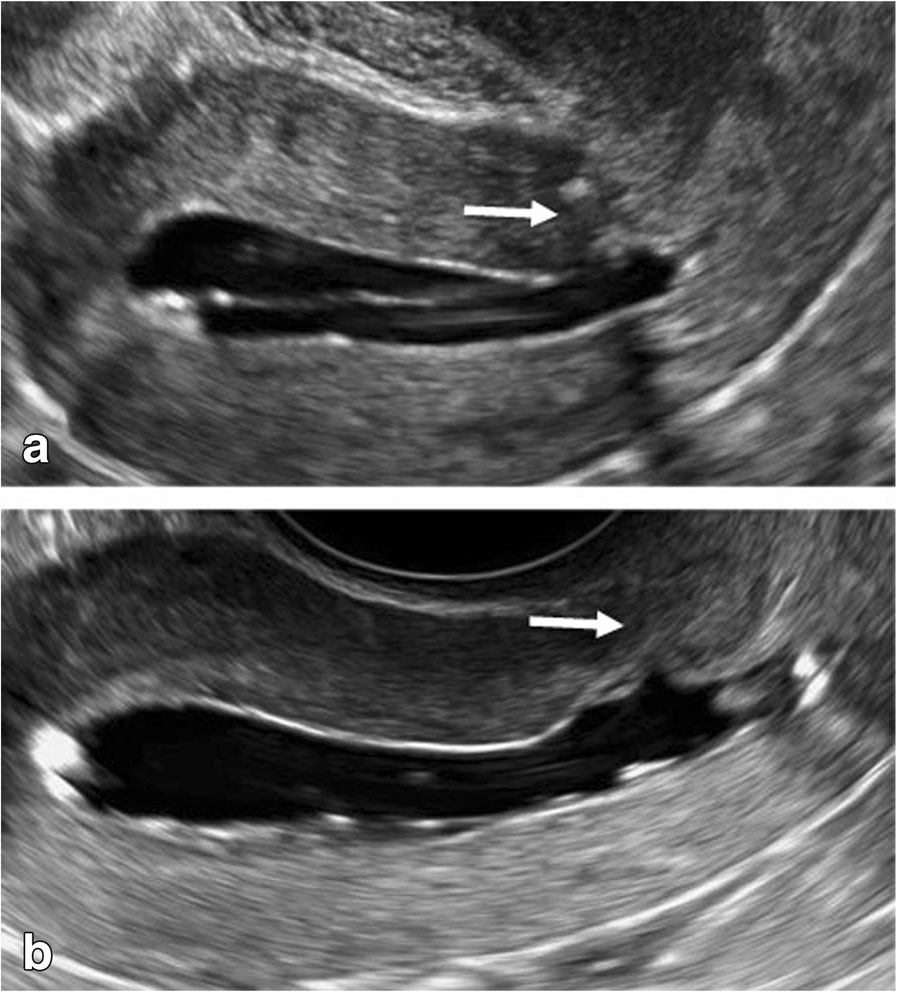
Saline contrast sonohysterography images. The *arrows* indicate the caesarean section scar 6 months after the index caesarean (**a**) and 6 months after the end of the heterotopic caesarean scar pregnancy by vaginal delivery (**b**). The thickness of the remaining myometrium appeared almost unchanged

We describe the case of a 27-year-old white woman who had experienced an emergency caesarean delivery at 39 weeks for fetal distress with no postpartum complications. As part of our ongoing study “Vaginal delivery after caesarean section”, she underwent saline contrast sonohysterography 6 months after the caesarean section. The caesarean scar had a small indentation and the remaining myometrium over the defect was 7.5 mm (Fig. 1a).

**Fig. 2:**
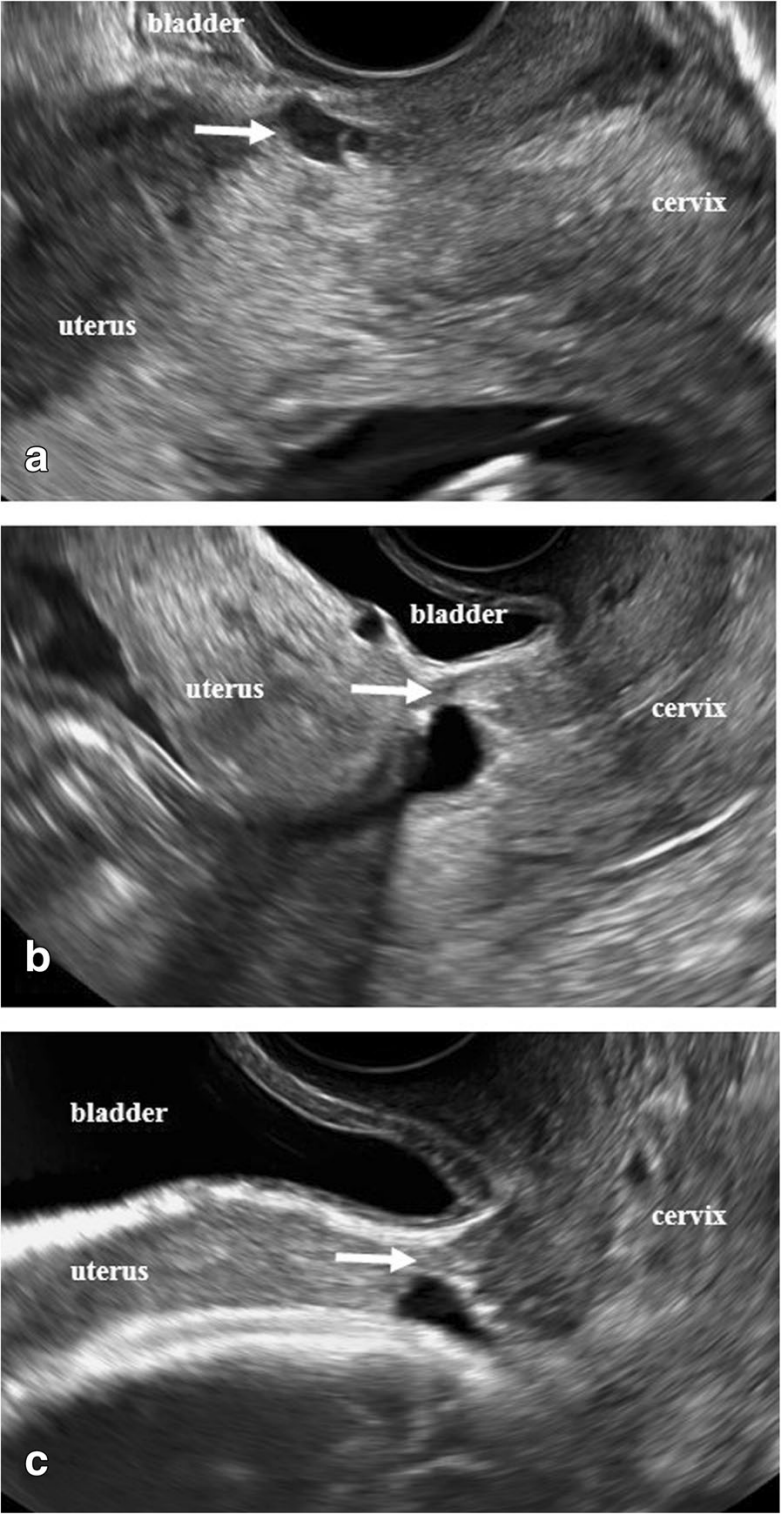
Transvaginal sonographic images. The *arrows* indicate the appearance of the cesarean scar area at the presence of the scar pregnancy at 13 + 2 (**a**) and after reabsorption of the scar pregnancy at 22 + 0 (**b**) and at 30 + 2 (**c**) weeks of gestation

In the current pregnancy, she had a dating scan at around 11 weeks with no remarks. She came for a transvaginal ultrasound examination at around 13 weeks as part of our study. This scan revealed a duplex pregnancy with one viable intrauterine fetus with normal anatomy and placenta located high on the anterior wall and a small gestational sac (8 mm) with a yolk sac without embryo was located in the caesarean scar (Fig. 2a). There was no extensive vascularity surrounding the sac. One corpus luteum was found in each of the two ovaries. She was asymptomatic.

She was informed that not enough evidence existed to advise a specific management of this condition. After discussion with her and her husband, expectant management was chosen with a new ultrasound examination after 5 weeks.

She came to our ultrasound department at 18 weeks, 22 weeks, and 30 weeks of gestation. She remained asymptomatic. The ectopic gestational sac was not visualized with transvaginal or transabdominal scans at the 18 weeks examination (Fig. 2b). The niche in the scar and the thickness of the thinnest part of the remaining myometrium appeared unchanged at all visits. The intrauterine pregnancy developed normally with no signs of abnormal placentation. At 30 weeks of gestation the ultrasound appearance of the scar area did not indicate any contraindications for vaginal delivery. The thickness of the lower uterine segment (LUS) was 4.9 mm (Fig. 2c). In agreement with our patient, vaginal delivery was planned. The staff of the labor ward was fully informed.

She was admitted to the labor ward with irregular contractions in week 37 + 0. Her cervix dilated to 3 cm with no further progress. Due to that oxytocin augmentation was administered for 3 hours. The duration of active labor was 6.5 hours. A healthy male neonate weighing 2985 g was delivered, with Apgar scores 9–10 at 1 and 5 minutes and umbilical cord pH 7.27. The placenta delivered spontaneously and total blood loss was 250 ml. The postpartum period was without any complications, and she was discharged home the next day.

At a follow-up visit 6 months postpartum, saline contrast sonohysterography showed no signs of the previous CSP, and the remaining myometrium over the hysterotomy scar defect was 5.7 mm (Fig. 1b).

Ethical approval for the ongoing study was obtained by the Ethics Committee of the Medical Faculty of Lund University, Sweden, reference number 2013/176. Our patient has given permission for publication of this case report in a scientific journal.

## Discussion and conclusions

Management of heterotopic CSP with an intrauterine gestation is a challenge. Treatment options for CSP include expectant management, and medical or surgical termination [1–4].

The use of methotrexate has been reported in management of ectopic gestations, but in heterotopic pregnancies with preservation of intrauterine pregnancy this may cause a teratogenic effect with fetal anomalies [5, 6].

A few case reports have described treatment of heterotopic CSP viable pregnancies with local injection of potassium chloride. This method is commonly used for fetal reduction in multiple pregnancy [7–9]. Treatment with potassium chloride is associated with an increased risk of abdominal pain, pregnancy loss, excessive vaginal bleeding, prematurity, need for subsequent surgery, and spontaneous rupture of membranes and subsequent chorioamnionitis [1, 8–12].

Laparoscopic treatment can be an option for removal of an ectopic scar pregnancy, but there is increased risk of hemorrhage and miscarriage [7, 13–15].

Michaels *et al.* suggested that expectant management can be appropriate in early gestations with no heartbeat, often resulting in complete absorption of the trophoblast [10]. Our patient had no bleeding or abdominal pain. The gestational sac located in the scar was non-viable with no extensive vascularity.

It is difficult to study possible changes in the tissues of the caesarean scar area after reabsorption of CSP. With ultrasound one can appreciate the thickness of LUS during the pregnancy, but not the quality of the myometrium.

Interestingly, it was found that our patient had a small defect in the uterine scar detected at ultrasound 6 months after caesarean section. Jurkovic *et al.* reported 18 cases of CSP over a 4-year period [1]. They observed that the majority of scars were well-healed. These data suggest that the size of a defect in the scar does not increase the risk of CSP; however, more studies are needed.

Our patient had a “normal” dating scan at 11 weeks. The early diagnosis of a heterotopic CSP is easy to miss, in particular with presence of an intrauterine viable embryo. Serum human chorionic gonadotropin (hCG) is of little value as long as an intrauterine pregnancy is ongoing. Transvaginal ultrasound is the best tool for diagnosis and management of such pregnancies. In our ongoing study “Vaginal delivery after caesarean section” we assess multiple parameters: scar area/scar pregnancy/potential placental complications. These ultrasound characteristics and clinical evaluation together with close monitoring provide support for the obstetrician in management of these women.

The literature is sparse and we still lack evidence and strong clinical guidelines to manage heterotopic pregnancies. Each woman diagnosed with scar implantation should receive an individual approach.

## Abbreviations

CSP: Caesarean scar pregnancy
hCG: Human chorionic gonadotropin
LUS: Lower uterine segment
